# Retired para athletes hold limited leadership roles in Canadian national sport federations

**DOI:** 10.3389/fspor.2025.1695467

**Published:** 2026-01-15

**Authors:** Ciara Stevenson, W. Ben Mortenson, Tzu-Hsuan Peng, Alexander Miniato, Elisabeth Walker-Young, David Willsie, Simone Gruenig, Andrea Bundon, Shawna Lawson, Mireille Landry, Courtney L. Pollock

**Affiliations:** 1 Department of Physical Therapy, University of British Columbia, Vancouver, BC, Canada; 2 Rehabilitation Research Program, Centre for Aging SMART, Vancouver Coastal Health Research Institute, Vancouver, BC, Canada; 3 Department of Occupational Science and Occupational Therapy, University of British Columbia, Vancouver, BC, Canada; 4 International Collaboration on Repair Discoveries, Vancouver, BC, Canada; 5 Consultant, Vancouver, BC, Canada; 6 Consultant, London, ON, Canada; 7 School of Kinesiology, University of British Columbia, Vancouver, BC, Canada; 8 Department of Physical Therapy, University of Toronto, Toronto, ON, Canada

**Keywords:** athlete career pathways, disability representation, para athletes, para sport, sport governance, sport management

## Abstract

Despite increasing support for Paralympic athletes in Canada, concerns have been raised about their limited involvement in leadership positions within the National Sport Federations (NSFs) that govern Paralympic sports in Canada. We sought to determine the representation of retired para athletes in leadership roles across Canadian NSFs, and identify strategies, facilitators and barriers to retaining para athlete expertise in these roles. An electronic survey was distributed via email to all 27 NSFs representing Canada's summer and winter Paralympic sports and total of 18 NSFs participated. Representation across all roles was found to be low, ranging from 5.29% in leadership positions, such as boards and committees, to 0.29% in classifier roles. Open-ended responses highlighted strategies for increasing para athlete inclusion in leadership roles after retirement from competition. Seven NSFs reported using formal and informal inclusion strategies. 10 NSFs relied solely on informal or reported no strategies. The most reported facilitator of inclusion was active recruitment, with ongoing communication between NSF leaders and retiring athletes. Conversely, the most reported barrier was the lack of representation of retired para athletes in leadership roles, which contributed to limited awareness of available opportunities. Currently, para sport in Canada is predominantly led and driven by non-disabled individuals, rather than the disabled individuals they purport to represent.

## 1 Introduction

Canada has a strong history of leadership in the Paralympic^1^ Movement. Canada led the proposed amalgamation and restructuring of four international disability specific sport organizations (visual impairment, amputees, cerebral palsy and spinal cord injuries) to collaborate with the International Olympic Committee (IOC), culminating in the creation of the International Paralympic Committee (IPC) in 1988 (1, 2). This leadership has been complimented by Canada's strong and sustained performance at the games. Canada first participated in the 1968 Tel Aviv Paralympic Games sending 25 athletes and winning 19 medals and ranking 12th overall (3). The nation has since participated in every Paralympic Games. Most recently at Paris 2024, Canada won 29 medals (10 gold, 9 silver and 10 bronze), ranking 12th among 86 participating countries (4, 5). Across various sports, Canada has decades of experience building sport-specific knowledge and capacity in athletes with disabilities.

In the early 2000s Canada transitioned from a parallel disability sport system to an integrated model, aligning policies, funding structures, and organizational mandates. While integration brought increased resources and recognition, it also introduced complexities around equity and representation (6). Under a new funding accountability framework, Sport Canada, the major federal funding agency for national team sports in Canada, declared their intent to stop funding “two systems” (one for disability sport and one for non-disability sport) and required non-disabled National Sport Federations (NSFs) to start delivering programs for persons with a disability and specifically to take on the responsibility for Paralympic pathways (i.e., the development of Paralympic athletes and the selection of teams to the Paralympic Games). These changes were implemented gradually, facilitated by funding incentives and accountability frameworks (7).

By the early 2000s, most para sports were governed within integrated NSF's, with exceptions for sports exclusive to athletes with disabilities (8). Integration models can vary within sport federations; however, in Canada, two broad models can be categorized. The first format is an integrated model with unified programs where the NSF is responsible for both non-disabled and para versions of the sport. The second format involved paralympic sports that do not have a non-disabled equivalent and remain governed by DSOs (e.g., Blind Sport Canada oversees the delivery of goalball, a sport exclusively for athletes with visual impairments) or non-integrated NSFs (e.g., Boccia Canada and Wheelchair Rugby Canada).

Although integration aimed to equalize resources and access, it also reduced para athletes from majority members in DSOs to minority voices within larger NSFs (6). Concerns persist about leadership and governance roles being dominated by non-disabled individuals (9). In sport, coaches, managers, chief executive officers (CEOs) and others in leadership roles all influence the people and directions of their organizations that influence sporting success (10).

Substantive representation in leadership is essential to ensure the needs and interests of diverse populations are reflected in practices and policies, thereby fostering more equitable and effective governance (11–14). In the context of leadership, women and ethnic minorities are sometimes described as “tokens” occupying a small minority position within a skewed group dominated by a large majority (13). Tokenism, however, is insufficient for creating and sustaining diversity in leadership or realizing its benefits in organizational culture, discourse and policy (15). In many cases, it can even reinforce existing organizational cultures and the privileges of the majority (13, 14). The theory of critical mass posits that a threshold of 30% of representatives of minority groups, such as people with disabilities, women or racial and ethnic minorities, etc. is necessary for their qualities and skills to be recognized rather than overlooked (16). The recognition that representative leadership constitutes substantive leadership with a greater capacity to effect change, combined with the historical and ongoing underrepresentation of minority groups and the persistent undervaluation of their leadership attributes, underscores the timeliness and importance of investigating the representation of people with disabilities in para sport leadership.

In para sport, organizational leadership is inherently multi-dimensional and includes coaches, high-performance directors, managers, classifiers, medical and support staff, and members of the National Paralympic Committee (NPC) (17). These actors are critical to shaping policy and practice from initial athlete attraction through to retirement (17). However, sport development processes often overlook athlete transitions to leadership roles, particularly for para athletes (18). Therefore, for this paper organizational leadership in sport refers to the strategic direction, governance, and policy implementation led by key contributors within the sport system, particularly those associated with NSFs, that influence athlete development and success across all phases of the athletic career.

In this study, leadership in Paralympic sport focuses on roles within NSFs, referring broadly to five categories: 1) leadership roles (commonly volunteer positions on boards, commissions, committees, etc. that establish policy, contribute to strategic decision making), 2) employee roles (paid positions such as CEOs, coordinators, sport managers, etc. responsible for organizational operations), 3) national team coach roles (assistant coach, head coach, etc.), 4) officials (umpires, referees, race officials, etc.) and, 5) classifiers (classifiers are trained experts such as physicians, physiotherapists, coaches, sport scientists, psychologists, and ophthalmologists, who assess the impact of impairments on sport specific function, and allocate a sport class for competition (19). While coaches, officials and classifiers may be paid employees of an NSF or volunteers, they are treated in this study as distinct and mutually exclusive leadership categories.

This work is positioned within Critical Disability Studies (CDS) which is a body of scholarship that seeks to challenge dominant social, cultural, and institutional norms that reinforce ableism and marginalize people with disabilities. CDS shifts the focus from individual impairments to the structural barriers, power imbalances, and systemic exclusions that shape opportunities for people with disabilities (20). Imbalance in leadership roles being held only by non-disabled individuals may perpetuate ableist norms, marginalize disabled voices, and result in decision-making that overlooks the needs of para athletes (21). Moreover, ableist norms can lead to policies and practices that, while appearing inclusive, actually maintain the marginalization of disabled individuals by upholding standards and norms that do not account for diverse experiences and needs (7). For example, supporting inclusion when it aligns with medal-winning potential or performance funding can lead to selective inclusion of athletes with minimal impairments or those that can quickly reach elite levels, marginalizing others (7). Involving individuals with disabilities in leadership roles has been recommended for developing policies and practices that genuinely reflect the needs and perspectives of the disabled community (22).

While there is robust research exploring what might be termed “Paralympic pathways” in Canadian sport systems, e.g., the routes into para sport and the career trajectories of para athletes (23–25), there remains a gap in terms of understanding to what extent persons with disabilities are involved in other roles within the national organizations. This is important to understand given that the Canadian Sport Policy outlines “Sport for Development”, as promoting “positive values at home and abroad” and developing individual athletes as leaders and role models in society generally and in sport specifically (26, 27). Although scholars have argued that para sport has historically been created *for* people with disabilities but led *by* non-disabled individuals, and that even in disability-specific sport contexts, people with disabilities are rarely included in leadership or decision-making roles (9), there is little research empirically examining participation in leadership and governance within Canada's sport system. This gap, along with concerns raised about the lack of diversity in disability sport administration (28–30), set the premise for our work. While extant research has examined disability representation in sport participation at both community and elite levels (24, 31, 32), it is unknown to what extent para athletes transition into leadership or decision-making positions within the Canadian sport system. Retired athletes may pursue leadership roles in their sport for various reasons, such as addressing a need within the federation or giving back to their community (33–35). Coaches with disability specific knowledge are perceived as highly influential throughout all phases of a para athlete's career (17, 36–38). However, little is known about the inclusion of retired para athletes within an NSF. To address this gap, we conducted a cross-sectional survey study to 1) determine the representation of retired para athletes in leadership roles within NSFs in Canada; and 2) gain insight into NSF staff perceptions regarding strategies, facilitators and barriers to retaining retired para athlete's expertise and participation in national sport administration and governance.

## 2 Methods

We used a cross-sectional survey to address our study objectives. It was designed based on the methodology outlined by Dillman et al. (39). The study was approved by the local university ethics board (H23-02105). The results of the survey are reported using the Checklist for Reporting Results of Internet E-surveys (CHERRIES) (40).

### 2.1 Data collection, sampling and respondents

The survey included 12 questions and 10 sub-questions. The survey included demographics questions including asking respondents about their sport, how long they have worked for the NSF, their role within the NSF, whether they are a retired para athlete from that sport and whether they have lived experience with disability. Respondents were asked to estimate the percentage of retired para athletes filling six administrative and governance roles within their NSF using a sliding scale from 0% to 100%. These roles included 1) leadership roles (boards, commissions, committees, etc.); for this paper leadership roles are defined as roles on boards, commissions or committees., 2) employee roles (coordinators, sport managers, etc.) 3) national team coach roles (assistant coach, head coach, etc.), 4) officials (umpires, referees, race officials, etc.), 5) classifiers, and 6) other roles.

Open-ended questions probed the strategies used by NSFs to retain retired para athletes in identified governance and administrative roles. Open-ended questions included: “1) Please describe your National Federation's strategy for inclusion of retired para athletes in your sport at the national level (roles such as technical officials/referees, classifiers, coaches, board, or commission members). Please also indicate when it was implemented. 2) What strategies do you use to ensure all athletes are aware of the available pathways for inclusion back into their sport after retirement? 3) If any, what are some perceived barriers to para athlete transition to national roles such as technical officials/referees, classifiers, coaches, board, or commission members?” And 4) If any, what are some perceived facilitators of transition of para athletes from their sport to the national roles such as technical officials/referees, classifiers, coaches, board, or commission members?”

The survey was pilot tested on four people previously involved in sport governance and administrative positions, including two retired Canadian Paralympians. The survey was administered using Qualtrics survey software (Provo, UT) and distributed via email to each of the Canadian coordinators of all 27 summer and winter paralympic sports with unique international governing bodies (41). Email addresses were sourced from NSF websites. All questions on the survey were voluntary. If a response was not received to the initial message, up to three bi-weekly reminder emails were sent. All data received from the survey was included in the analysis. Four of the 18 responses were incomplete. The quantitative responses for these four were all complete and included in the analysis in Table 2. No qualitative responses were received and were therefore not included in Tables 3–5. The four incomplete responses did not include demographic data which is noted in Table 1.

**Table 1:** Characteristics of organizational respondents that completed the survey.

Characteristic category	Number	%
How long have you been involved with the National Federation?		
<3 years	3	16.7
3–5 years	3	16.7
5–10 years	1	5.6
10–20 years	1	5.6
>20 years	6	33.3
Unanswered	4	22.2
Are you a retired para athlete from this sport?		
Yes	4	22.2
No	10	55.6
Unanswered	4	22.2
What is your age?		
20–30	1	5.6
31–40	4	22.2
41–50	3	16.7
>50	6	33.3
Unanswered	4	22.2
To which gender identity do you most identify?		
Women	4	22.2
Men	8	44.4
Prefer Not to Answer	2	11.1
Unanswered	4	22.2
Do you identify as being non-disabled, or having lived experience with a disability?		
Lived experience with a disability	4	22.2
Non-disabled	10	55.6
Unanswered	4	22.2

**Table 2:** An estimate of the percentage of retired para athletes that hold various roles within each national federation*.*

Organizational role	Average % (SD)	Min %	Max %	Number of sports with >0%
Officiating Roles (umpires, refs, race officials, etc.)	0.83 (1.46)	0	5	6
Classifier roles	0.28 (0.73)	0	3	3
Employee roles (coordinators, sports managers, etc.)	1.72 (2.18)	0	7	9
Leadership roles (boards, commissions, committee, etc.)	5.17 (7.52)	0	30	15
National team coach roles (assistant coach, head coach, etc.)	2.22 (3.61)	0	10	7
Other roles	2.11 (4.41)	0	15	5

**Table 3:** Strategies used by NSFs to recruit and retain para athletes in governance and administrative roles following retirement and the number of times each strategy is reported. Eleven NSFs reported between one and three strategies. The number of instances each strategy was described is reported.

Informal strategies	Number of reports (of 11 responses)
Actively trying to recruit through informal personal communication	7
Athlete council to promote early involvement in governance	1
Identification of additional resources external to the NSF	1
Formal strategies	Number of reports (of 11 responses)
Offboarding packages for athletes including engagement opportunities	2
Accommodation for employment opportunities	1
Digital promotion of opportunities	1
Lobbying for funding to support the creation of new roles	1
Retirement planning with athletes	1

**Table 4:** Estimates of the percentage of retired para athletes that hold various roles within NSF's that reported using at least one formal recruiting strategy (*n* = 11) compared to those that used informal strategies (*n* = 7). *P* values were calculated using a two tailed independent *t*-test*.*

Organizational role	Average of sports with at least one formal strategy (%) *n* = 11	Average of sports with informal strategies (%) *n* = 7	*p* Value
Official roles (umpires, refs, race officials, etc.)	1.00	0.73	0.72
Classifier roles	0.29	0.27	0.97
Employee roles (coordinators, sports managers, etc.)	2.28	1.45	0.54
Leadership roles (boards, commissions, committee, etc.)	2.71	6.73	0.30
National team coach role (assistant coach, head coach, etc.)	1.43	2.73	0.49
Other roles	0.00	3.45	0.12

**Table 5:** Perceived barriers to inclusion and incidence at which they were reported. Respondents from 14 NSFs reported between one and three perceived barriers, all of which have been reported.

Barrier	Number of reports (of 14 responses)
Not seeing people like themselves in these leadership positions	3
Impairments posing challenges to taking on certain roles	3
Lack of desire to coach	2
Retiring athletes relocate away from centralized NSF	2
Limited opportunities within an NSF	2
A perception that para athletes cannot take on roles that are not para specific	1
A lack of desire of NSFs to create space for athletes	1
Skills required to perform the role	1

### 2.2 Data analysis

For the quantitative data, average, minimum and maximum percentage values were calculated. To provide context, the number of sports that reported an estimated percentage greater than zero for each role is also reported. For the seven sports that identified using at least one formal strategy to recruit and retain retired para athletes in governance and administrative roles, the average percentages for representation by retired para athletes in official, classifier, employee, leadership, coaching and other roles were calculated. The difference between the average estimates was compared to that of the 11 sports that did not report using any formal strategies to recruit and retain para athletes in administrative and governance roles.

Content analysis was conducted to identify specific sets of words and concepts within respondents' written responses (42). The survey responses were selectively reduced to a set of words and phrases that answer the three main questions presented in the survey. Reported responses addressing national federation's strategy for inclusion were categorized as either *formal strategies* for inclusion, defined as structured initiatives designed and implemented to ensure retiring para athletes have opportunities to participate and succeed in administrative and governance roles in their NSF's or, *informal strategies* defined as involving everyday communication, interactions and culture to contribute to an inclusive NSF. A two-tailed independent *t*-test was used to test for differences in reported percentage of retired para athlete representation in each role between NSFs that reported at least one formal strategy and NSFs that reported only informal strategies, significance set at *p* < 0.05. Person first vs. identity first language is used interchangeably based on the respondent's choice of language.

## 3 Results

### 3.1 Respondents

Survey responses were received from 18 of 27 NSFs contacted. 9 NSF's did not respond to the survey. Fifteen of 22 summer Paralympic sports responded. Four of six winter sports responded. One participating NSF governs two sports that are represented by two distinct international governing organizations. We have chosen to consider them as two sports since the NSF and governance is bifurcated between the sports. We have not reported the sport each respondent represents to preserve their anonymity. Of the 18 responses, 14 completed the survey in its entirety, four sports responses were incomplete. The four incomplete responses included quantitative results estimating the percentage of retired para athletes in each designated governance and administrative role. None of the incomplete responses included answers to any of the open-ended questions or demographic questions. Data from all 18 participating NSFs, including four incomplete surveys, were included in the quantitative analysis.

### 3.2 Respondent demographics

In Table 3 we report on the characteristics of the respondents that completed the survey. One third of respondents had been involved for more than 20 years while nearly over one fifth of respondents have lived experience with disability. Notably, the four respondents that reported a lived experience with disability are the same four that are retired para athletes from the sport they are representing.

### 3.3 Representation by retired para athletes in governance and administrative roles within Canadian NSFs

The average reported percentage of retired para athletes in officiating, classifier, employee, leadership, coaching and other roles was low (Table 2) with the highest representation in leadership roles and the lowest in classifier roles. Every role had retired para-athletes filling that role in at least three sports and up to 15 sports. The lowest estimated percentage of representation reported by an NSF [Min (%)] was 0% amongst all roles and the largest [Max (%)] was 30% in leadership roles. Of the 15 sports that had representation of more than 0%, twelve sports reported less than the average (5.17%) representation in leadership roles and three sports estimated 0% participation by retired para athletes in leadership roles.

Of the 18 NSFs that responded, half reported some level of inclusion of retired para athletes in employee roles such as coordinators and managers. Despite this, overall participation in these positions was low. Seven NSFs indicated that retired para athletes were involved in coaching at the national team level yet these individuals made up a very small percentage of all national team coaches. A similar trend was observed in officiating roles. Additionally, five NSFs noted representation of retired para athletes in “other” roles. One of these was specified as a legal position, and another as a development ambassador. Two NSFs referred to roles as provincial or club coaches, which fall outside the scope of this study. One NSF did not provide clarification on the nature of the “other” role.

### 3.4 Canadian NSF's strategies for inclusion of retired para athletes in governance and administrative roles

11 NSFs responded to the open-ended question asking about the strategies used to ensure all athletes are aware of the available pathways for inclusion back into their sport after retirement. Eight unique strategies were identified as indicated in Table 3. Three organizations reported using no formal strategy. Representative quotes from respondents were identified to illustrate reported strategies. The most common strategy used by seven respondents NSF was actively trying to recruit through informal personal communication. This involved actively recruiting retired para athletes and encouraging them directly to apply for positions through informal personal contact and communication with the athlete. “Informal strategies” were commonly reported and one respondent who identifies as a retired para athlete indicated, “there [was] no formal strategy” and “people [were] hand-picked or pursue[d the] opportunity of their own accord”. One respondent reported providing, “one-on-one discussions to look at options and where they are at individually in their education and career goals”.

Retirement planning as a formal strategy for inclusion was described by respondents as the NSF providing information to retiring para athletes on opportunities to stay involved with the organization beyond participation as an athlete. NSF driven strategies included exit interviews and offboarding packages, promoting coach education for retiring athletes such as National Coaching Certification Program (NCCP) training. Creating mentorship opportunities was also a strategy employed by two NSFs to promote the inclusion of retired para athletes at the national level in governance and administrative roles. Another reported strategy is assisting athletes with retirement planning. A respondent who is a retired para athlete reports that their NSF provides an “offboarding package for athletes.” NSFs assist retiring athletes with their transition to coaching by allowing them to “advance coaching certification quickly”. Athletes are also directed towards Game Plan resources—a Canadian national program that supports national team athletes' holistic wellness at all stages of their Olympic or Paralympic journeys, including retirement (43). Another respondent reported that, “athletes receive an initial info package with retirement resources that includes these engagement opportunities and further individual engagement specific to volunteer and coaching opportunities.” Two NSFs reported offering an offboarding package for athletes upon retirement which includes engagement opportunities. Additional reported strategies included building upon disability accommodations to work in the NSF (e.g., assistive technology), an athlete council to promote more athlete involvement in governance prior to retirement and more direct communication with the board of directors and retirement planning with athletes.

Our data shows that most NSFs (11/18) currently do not employ a formal strategy to retain para athletes in governance and administrative roles post-retirement. When efforts are made, they typically occur on a case-by-case basis, working directly with retiring athletes and using one-on-one conversations to guide their pathways. Seven of the 18 NSFs reported implementing at least one specific, formal strategy to increase the inclusion of retired para athletes in governance and administrative roles. In Table 4 we report the estimated percentages of retired para athletes in specific governance and administrative roles for NSFs with formal and informal strategies. There were small but statistically insignificant differences between NSFs with formal strategies and NSFs with informal strategies in reported percentages of inclusion of retired para athletes in any role.

### 3.5 Barriers to including retired para athletes in national governance and administrative roles

Fourteen NSFs responded to the question asking about the barriers faced by athletes to pursuing governance and administrative roles upon retirement. Table 5 indicates the barriers reported, from most reported to least. Eight unique barriers were reported and two NSFs reported “none”. Within the barrier, “not seeing people like themselves in these leadership positions”, one respondent reported that there was “a perception that [the para version of the sport] may not be seen as being as central or high-profile to the NSFs operations may discourage application/invitation to board/commission membership.” A respondent that identified as a retired para athlete highlighted the barrier that many retiring para athletes were unaware of available positions within the NSF. They state, “representation matters,” noting that without visible diversity within the NSF, many para athletes might not consider roles within NSFs to be a possibility. Another retired para athlete felt that there is “no desire from the NSF to start programs or make space for athletes, even if a desire to be involved is expressed.”

Physical impairments were also reported as a barrier to people with disabilities performing certain roles within an NSF. It was specifically noted that “for referees, for example, people with disabilities are generally not considered for this role except in very grassroots programming”. Travel considerations (e.g., driving a rental car) were also suggested as a barrier for people with disabilities acting in a support role for an athlete with a disability. A respondent who is also a retired para athlete noted that the “physical realities of day-to-day coaching” in their sport can present additional challenges. Another non-disabled respondent reported example is, “Visual impairment [being] a major challenge for Paralympic [sport). With today's level of technological support available, some of the visually impaired can act in a coaching capacity, however, they need a sighted assistant at all times. Refereeing is not an option for a visually impaired person.”

Additional barriers reported were a lack of desire to coach, retiring athletes move away from NSF/training center region to be close to family/home and limited opportunities within NSFs. Additionally, it was discussed that there is “little turnover” in employee and coaching and official roles. Another reported barrier is the perception that retired para athletes cannot take on roles that are not para specific such as taking on governance and administrative roles within the NSF that are affiliated with the non-disabled or non-para sport programs.

### 3.6 Facilitators for including retired para athletes in national governance and administrative roles

Thirteen NSFs responded to the question asking about the facilitators for inclusion of athletes in leadership and governance roles upon retirement. Table 6 relates all the perceived facilitators to inclusion that were reported. Eight unique facilitators were reported and three NSF's reported “none”. The most reported facilitator was active recruitment, by means of continued contact between relevant practitioners and leaders in the NSF and athletes as they retire. Notably this was reported as an informal strategy for inclusion as well. This was seen as valuable in retaining the expertise of retiring para athletes. Additionally, NSFs identified a need to have communication between para athletes and those in governance and administrative roles prior to athlete retirement. One respondent suggested that a way to increase involvement would be to provide current athletes with opportunities to participate in making decisions particularly those that are directly related to their program. This could involve “having the option to participate on the board or on committees”. Another respondent also identified the need to “get [athletes] involved earlier before they retire” and suggested looking at options for “athletes’ council or other committees [where] they are active players”. It was suggested that working with athletes prior to retirement and on retirement planning allows athletes to understand what opportunities are available to them in a governance and administrative capacity.

**Table 6:** Perceived facilitators to inclusion and the incidence at which they were reported. Respondents from 13 NSFs reported between one and three perceived facilitators, all of which have been reported*.*

Facilitators	Number of reports (of 13 responses)
Active recruitment strategies	4
Options to participate in boards and committees before retirement	3
Education and mentorship resources	3
A champion of para sport within the NSF	2
Exit interviews and transition	1
External assistance	1
Not limiting the number of coaches even if athlete participation is low	1
Interest by athletes matching the needs of the federation	1

Retirement planning was a facilitator to athlete recruitment into administrative and governance roles. Practical examples included, an “exit interview” to discuss “transition plans” as noted by one NSF. One retired para athlete suggested resources external to the NSF such as Game Plan and “active recruitment for positions” may be necessary to facilitate the inclusion of more retiring para athletes in roles within NSFs. Respondents also highlighted the provision of education opportunities to give retiring athletes the skills and qualifications to pursue governance and administrative roles as a facilitator to retain this knowledge base. Examples included providing financial support to obtain certifications and NCCP training and mentorship resources to help facilitate athletes taking on coaching roles that they would otherwise be unqualified for.

Three of the four respondents with lived experience in para sport believed it was essential to have someone within the NSF who values and champions para sport, as this promotes awareness of opportunities within the sport. One retired para athlete emphasized, with what could be perceived as a level of frustration, the importance of, “having someone within a national federation who actually gives a sh*t about para”. Another stresses the need for “both sides seeing the value in the other” i.e., the NSF and the athletes. Increasing representation requires both “interest by the athlete and a need within the federation.”

## 4 Discussion

Guided by CDS, which conceptualizes disability as a social, political and structural phenomenon rather than as an individual deficit, findings are interpreted in relation to power, representation and governance within national sport systems. In line with CDS, there has been a growing global commitment to enhance both the visibility of, and active engagement among people with disabilities in society and sport (22, 44–45). As of 2022, nine of the 14 members (64%) of the IPC's Governing Board were Paralympians or para athletes and by 2023, 16% of the IPC's workforce identified as having a disability (46, 47). These figures suggest a growing commitment to disability representation within the IPC's leadership and organizational structure. In Canada, the disability rate has risen 4.9% between 2017 and 2022 to 27.0%, with a rate of 24.1% amongst working age individuals between the ages 25–64 (48). This study aimed to examine the representation of retired para athletes in governance and administrative roles within NSFs in Canada.

### 4.1 Representation by retired para athletes in NSF leadership roles

This is the first study to examine the representation of and strategies to retain retired para athletes in governance and administrative roles within Canadian NSFs. Critical mass theory suggests that once minority members reach a 30% threshold, they can shift from being observers to active participants, thereby influencing the organizational culture and policies more effectively (49). None of the 18 Canadian NSF's that responded to the survey reached this threshold, meaning the experiences of retired para athletes may not be adequately reflected in decision making and strategic leadership in organizations formed to govern both Olympic and Paralympic streams of sport in Canada. This lack of representation may impede the influence retired para athletes can exert on the para sport system to meet future needs of athletes with a physical disability striving for success in sport. Lived experiences of disability is necessary to understand how the decisions, actions, and policies of the NSF will impact disabled athletes lives. Consistent with CDS, representation in this context is understood not merely as numerical, but as a means of redistributing power and shaping organization priorities in ways that reflect lived experiences of disability.

The highest estimated representation of retired para athletes was in typically unpaid roles, such as board, committee, or commissions at only 5.17%. The lowest representation was in classification roles, which may be due to the limited number of classifier positions and reliance on international classifiers in some sports. As classification is commonly a process completed by clinicians (e.g., physiotherapists, physicians, optometrists) this finding may also reflect systemic barriers that exist to accessing higher education including ableist culture, non-inclusive technical standards that don't account for accommodations, and a lack of clear and accessible information about disability support (50, 51). This may also be a factor that underpins challenges of the pathway of retired para athletes obtaining leadership and governance roles more broadly.

The literature highlights a clear connection between board composition and organizational performance, since boards guide strategic direction and shape culture within the organization (52, 53). Gender diversity is the most robust example of examining diversity and inclusion in sport governance literature (54). As a result of this work, countries such as Norway have introduced gender quotas to equalize representation in the Norwegian Olympic and Paralympic Committee and Confederation of Sport (55). Women bring unique skills, knowledge and perspectives to board level decision making, and gender diverse boards typically outperform those with single gender boards (49, 56). Drawing on this, it can be argued that Canadian NSF's, with an average of just 5.17% representation of retired para athletes in leadership has not achieved critical mass and are not positioned to perform optimally or make fully effective strategic decisions for para sport. There are no examples in current scholarly literature quantifying the representation of retired para athletes in roles of leadership. However, the United States Olympic and Paralympic Committee (USOPC) demographic report discloses a five-year average of 5.87% disability representation between 2020 and 2025 (amongst executive/senior level officials and managers, first and mid-level officials and managers, professional staff, technicians, administrative support workers, craft workers, operatives, and service workers) (57). It is unclear if this representation is inclusive of retired para athletes.

Coaches have been identified as a key role for supporting and influencing para athlete's development. However, despite coaching being a frontline administrative role involving direct athlete interaction, at 2.22% representation, very few national team coaches were reported to be retired para athletes. Congruent with our results, para sport coaches are typically non-disabled individuals with previous experience in non-disabled sport despite the unique demands of coaching para sport (58). These coaches often report a limited understanding of expectations when coaching athletes with disabilities and require structures of support that provide education, learning, and qualifications to gain disability specific knowledge (58, 59). In contrast, coaches with lived experience as para athletes offer unique and valued insights and disability-specific knowledge (58, 60). Disability-specific knowledge, in this context, refers to an understanding of how physical and/or sensory impairments affect functional abilities in training and sport performance (37). Providing appropriate training and support was identified by respondents as a key facilitator for increasing para athlete inclusion in coaching roles which would allow for para athletes to gain coaching specific skills to complement their sport specific skills and disability specific knowledge. Moreover, athletes with more severe impairments face greater challenges accessing sporting opportunities and accessing coaches with disability specific knowledge that can provide suitable training programs tailored to their needs (17, 61, 62). Despite evidence supporting the value of lived experience and disability-specific knowledge in coaching, less than half of NSFs reported having any retired para athletes in coaching roles. Therefore, there is a need to develop para sport governance structure in parallel with coach development pathways.

It was expressed that retired para athletes who wish to coach should not be restricted to para sport. This suggests retired para athletes should be considered for opportunities coaching both para athletes and non-disabled athletes in their sport. Restricting retired para athletes to coaching only in para sport, while non-disabled coaches can work across programs, has previously been identified as a barrier to equitable coaching opportunities (63).

### 4.2 Perception of strategies, facilitators and barriers to para athlete recruitment and retention

A commonly cited facilitator was support from external organizations and resources, particularly Game Plan, a national program funded by the Canadian Olympic Committee (COC) and the Canadian Paralympic Committee (CPC). Game Plan provides resources for athlete well-being, education, and career planning during and after athletic careers (43). It was established in response to needs identified in the 2010 Olympic Games Debrief Report and the 2011 Business Report, making it a relatively new program (63). Its long-term impact may not yet be fully realized, as retired and retiring para athletes may start in local management roles before progressing to national-level opportunities. However, our results suggest that to date, the impact has been minimal.

Internationally, there is a paucity of literature proposing effective strategies to address retired para athlete representation in sport governance and leadership. In the United Kingdom, it is reported that para athletes often face discrimination related to their disabilities when seeking employment (33) and employment training tailored for para athletes remains limited (63). Furthermore, in contrast to their non-disabled counterparts in the United Kingdom, para athletes have fewer opportunities to secure post-retirement roles in sport (63). Our results support this, suggesting that the lack of representation in NSF governance may hinder the career aspirations of retiring para athletes in sport-related roles in Canada citing disability as a factor despite CDS positioning disability as a social and political issue (20). Globally efforts are beginning to emerge to improve EDI (Equity, Diversity, and Inclusion) in elite sport leadership (64). More formal EDI mandates, such as equity targets and strategic diversity recruitment may be required from Sport Canada to counter these dynamics and increase representation within NSFs. Moving forward, there is a need to study the effectiveness of formal and informal strategies employed to increase the representation of retired para athletes in roles in the NSF.

Additional concerns remain regarding the perceived lack of initiative from NSFs in creating roles for retiring para athletes. Consistent with this perception, a frequently reported facilitator was the presence of an advocate or “champion” within the NSF who prioritizes para sport and values para athlete involvement. Until critical mass in leadership is achieved in Canada, Canadian para sport development depends on individual champions rather than being embedded within organizational culture. Prior research has identified such advocacy is critical, indicating that para athlete retention is not yet embedded in the sport system's culture as it is for non-disabled athletes (63). This highlights the ongoing marginalization of people with disabilities in sport.

While para sport aims to remove barriers to participation, physical and structural obstacles persist in governance and administrative contexts. Physical impairments were reported as barriers to inclusion, particularly in roles requiring physical demands, such as officiating and traveling for coaching duties. Requirements like driving a rental car at away competitions, though not inherently essential to coaching, are often treated as job prerequisites. In other employment sectors, such conditions could be subject to legal scrutiny and deemed discriminatory if not clearly outlined in the job description or addressed through reasonable accommodations (65, 66). These tasks could instead be reframed as a non-essential job functions, with organizations providing alternatives such as accessible shuttles and designated drivers. Furthermore, use of remote participation technology (e.g., zoom) could foster participation in governance without relocation. The latter has already become normalized in many workplaces after the COVID-19 pandemic and substantially reduces travel demands. At the policy level, national sport organizations could explicitly articulate what constitutes an “essential” job requirement and ensure compliance with broader disability legislation that mandates reasonable accommodations. The normalization and expectation of performing non-essential job functions in sport raises broader questions about what constitutes essential job functions vs. organizational convenience.

Although a goal of para sport is to challenge discrimination against individuals with physical disabilities while fostering positive social awareness and inclusion, it is not immune to ableist ideologies and exclusionary practices (29, 67, 68). Historically, the Paralympic movement was primarily led by non-disabled individuals, based on the assumption that expertise from non-disabled sport was necessary to establish para sport (29, 69). Given the growing number of retired para athletes with extensive experience in high-level competition, one might expect a shift in leadership demographics. Our data revealed a barrier to obtaining leadership roles was that athletes were not seeing themselves in leadership positions which implies a lack of representation leading to decreased knowledge of roles that could be sought-after opportunities for para-athletes. This suggests that additional mechanisms may be required to support the transition of retired para athletes into NSF roles irrespective of whether the role is specific to the disabled or non-disabled facet of the sport. Addressing ableism in sport requires ensuring diverse representation of para athletes at all levels, including governance, management, and coaching (29, 68). In sport administration and governance, it is increasingly important for individuals with disabilities to take on active leadership roles in shaping para sport culture, rather than merely being recipients of services (29).

From a CDS perspective, the persistent underrepresentation of para athletes in leadership reflects the reproduction of ableist power relations that privilege non-disabled leaders/administrators and career trajectories in sport governance. Rather than indicating a lack of interest or preparedness amongst para athletes, these patterns point to structural conditions that limit access and position disabled participants as passive participants in the sport system rather than knowledge holders and decisions makers. Our findings reveal that critical mass has yet to be established, therefore, future research should explore the effectiveness of implementation of a governance model that seeks to establish critical mass with respect to integration of retired para athletes into decision making structures for a more representative para sport system in Canada. Accordingly, future research should consider; 1) the utility of mandated equity targets for retired para athletes within leadership, governance and coaching roles, and 2) targeted capacity building with structured pathways for para athletes to transition to administrative and technical positions through mentorship, professional development, and accessible education. The goal is to move beyond *ad hoc* inclusion towards a para athlete-led governance paradigm that meaningfully aligns policy, representation and organizational performance.

### 4.3 Limitations and future directions

This study has four main limitations. First, the response rate was 64%, leaving nine Paralympic sports unrepresented in the data. A non-response, despite repeated invitations and flexibility in choosing a respondent, might indicate a potential reluctance or lack of organizational capacity to engage in this line of inquiry. Second, the data relies on a single respondent, which may not fully capture all organizational perspectives and results were not validated against organizational records. However, it is important to note that respondents were employees of the NSFs who are typically knowledgeable regarding roles within NSFs and their compositions. Role representation was estimated using a sliding scale defaulting to 0%. Responses of 0% were assumed to indicate no representation, not non-response when additional responses were provided. Third, representatives from NSFs were asked to report their perception of strategies, facilitators and barriers to inclusion which creates potential for social desirability bias. Qualitative survey responses were generally brief and the inability to ask follow-up questions limited the analysis to descriptive reporting. Finally, this study focused on national-level representation and did not capture participation by retired para athletes at local or provincial levels. In the survey, three NSFs reported that retired national team para athletes are working as club or provincial coaches. While this represents an important form of participation, future work is needed to fully elucidate the representation of retired para athletes in all levels of sports governance and administration. Moreover, to support greater inclusion of retired para athletes in coaching and management roles, future research is needed to critically explore solutions such as role differentiation, accessibility, modifications and workplace accommodations.

## 5 Conclusion

Leadership roles in para sport in Canada are largely occupied by non-disabled individuals; with minimal inclusion of the para athletes it represents. While some NSFs have introduced strategies to engage retired para athletes, reported percentages suggest that efforts remain inconsistent and insufficient. This study advances CDS through its integration with critical mass theory to explore how the underrepresentation of retired para athletes in national sport governance may reflect ableist institutional structures that limit representative leadership and redistribution of decision-making power. From a sport policy perspective, these findings suggest that voluntary inclusion efforts are insufficient and that formal governance mechanisms, such as mandated equity targets, accessible leadership pathways, and accountability measures, may be required to disrupt ableist structures and achieve representative decision making within national sport organizations. The findings that “informal communication” and “active recruitment” are the primary drivers of inclusion, combined with the stated need for an internal “champion”, suggests that current progress is reliant on individual advocacy rather than on robust, formal, and systemic policies required for sustainable change. More robust strategies, inclusive policies and further research are essential to building a truly representative and inclusive para sport system in Canada.

## Data Availability

The raw data supporting the conclusions of this article will be made available by the authors, without undue reservation.

## Footnotes

^1^In this paper, the terminology relating to disability and sport come from the IPC’s Guide to Para and IPC Terminology (1).
