# Supplementary material for: Retired para athletes hold limited leadership roles in Canadian national sport federations
Source: Front Sports Act Living. 2026 Jan 15;7:1695467. doi: 10.3389/fspor.2025.1695467 (PMC12852403; doi:10.3389/fspor.2025.1695467)
Supplement: Supplementary file 1 [file Datasheet1.pdf]

Survey: Retired Para Athlete Leadership in Canadian NSF

Q1. Please Identify your sport.

Text Box

Q2_1. Please use the sliding scale to estimate the percentage of retired para athletes that are in the following roles within your National Federation: - Official Roles (umpires, refs, race officials, etc...)

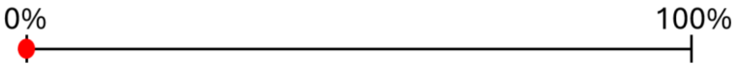

Q2_2. Please use the sliding scale to estimate the percentage of retired para athletes that are in the following roles within your National Federation: - Classifier role

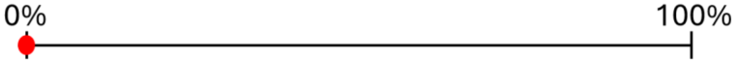

Q2_3. Please use the sliding scale to estimate the percentage of retired para athletes that are in the following roles within your National Federation: - Employee role (coordinators, sports managers, ect...)

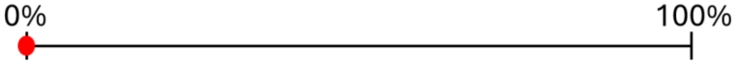

Q2_4. Please use the sliding scale to estimate the percentage of retired para athletes that are in the following roles within your National Federation: - Leadership role (boards, commissions, committee, etc...)

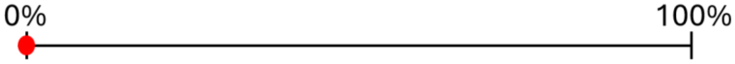

Q2_5. Please use the sliding scale to estimate the percentage of retired para athletes that are in the following roles within your National Federation: - National team coach role (assistant coach, head coach, etc...)

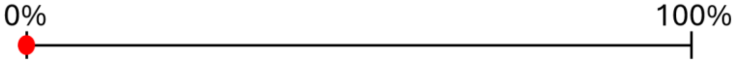

Q2_6. Please use the sliding scale to estimate the percentage of retired para athletes that are in the following roles within your National Federation: - Other (please specify)

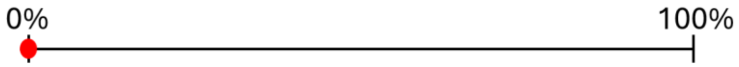

Text box

Q3. Does your National Federation promote the inclusion of retired athletes into the roles discussed previously (e.g. formal or informal retiring athlete pathway or other programs to support)?

- ○ Yes
- ○ No

Q4. Please describe your National Federation's strategy for inclusion of retired para athletes in your sport at the national level (roles such as technical officials/referees, classifiers, coaches, board or commission members). Please also indicate when it was implemented.

Text Box

Q5. What strategies do you use to ensure all athletes are aware of the available pathways for inclusion back into their sport after retirement?

Text Box

Q6. If any, what are some perceived barriers to para athlete transition to national roles such as technical officials/referees, classifiers, coaches, board or commission members?

Text Box

Q7. If any, what are some perceived facilitators of transition of para athletes from their sport to the national roles such as technical officials/referees, classifiers, coaches, board or commission members?

Text Box

Q8-a. What is your role in the National Federation?

Text Box

Q8-b. How long have you been involved with the National Federation?

- ○ <3 years
- ○ 3 to 5 years
- ○ 5 to 10 years
- ○ 10 to 20 years
- ○ >20 years

Q9. Are you a retired para athlete from this sport?

- o Yes
- o No

Q10. What is your age?

- o 20-30
- o 31-40
- o 41-50
- o >50

Q11. To which gender identity do you most identify

- o Male
- o Female
- o Prefer Not to Answer
- o Other: **text box**

Q12. Do you identify as being non-disabled, or having lived experience with a disability?

- o Non-disabled
- o Lived experience with a disability
